# Species Based Synonymous Codon Usage in Fusion Protein Gene of Newcastle Disease Virus

**DOI:** 10.1371/journal.pone.0114754

**Published:** 2014-12-05

**Authors:** Chandra Shekhar Kumar, Sachin Kumar

**Affiliations:** Department of Biotechnology, Indian Institute of Technology Guwahati, Guwahati, Assam, India; University of Athens, Medical School, Greece

## Abstract

Newcastle disease is highly pathogenic to poultry and many other avian species. However, the Newcastle disease virus (NDV) has also been reported from many non-avian species. The NDV fusion protein (F) is a major determinant of its pathogenicity and virulence. The functionalities of F gene have been explored for the development of vaccine and diagnostics against NDV. Although the F protein is well studied but the codon usage and its nucleotide composition from NDV isolated from different species have not yet been explored. In present study, we have analyzed the factors responsible for the determination of codon usage in NDV isolated from four major avian host species. The F gene of NDV is analyzed for its base composition and its correlation with the bias in codon usage. Our result showed that random mutational pressure is responsible for codon usage bias in F protein of NDV isolates. Aromaticity, GC3s, and aliphatic index were not found responsible for species based synonymous codon usage bias in F gene of NDV. Moreover, the low amount of codon usage bias and expression level was further confirmed by a low CAI value. The phylogenetic analysis of isolates was found in corroboration with the relatedness of species based on codon usage bias. The relationship between the host species and the NDV isolates from the host does not represent a significant correlation in our study. The present study provides a basic understanding of the mechanism involved in codon usage among species.

## Introduction

Newcastle disease virus (NDV) has been isolated from the various avian species around the world. Newcastle disease can result in severe economic losses to the poultry industry worldwide. NDV belongs to the genus *Avulavirus* under the family *Paramyxoviridae* [1]. However, NDV has been isolated from different non avian species [2]. Complete and partial genome sequences of NDV isolated from different species are being regularly reported from different parts of the world. NDV genome encodes six different proteins in order of a nucleoprotein (N), a phosphoprotein (P), a matrix protein (M), a fusion protein (F), an attachment protein called the hemagglutinin–neuraminidase (HN), and a large polymeraseprotein (L) from 3′-N-P-M-F-HN-L-5′ direction. The envelope of NDV contains two surface glycoproteins, the HN and the F protein. Various studies have shown that the amino acid sequence at the F protein cleavage site is a key determinant of NDV virulence [3], [4], [5], [6]. However, the cleavage of F protein in a wide range of host tissues is responsible for the systemic spread of NDV and also for its virulence [5]. The F protein being a surface glycoprotein is present on the NDV envelope and mediates its fusion with the host cell membrane. Furthermore, the F protein is assisted in its function by the HN protein and the productive infection of NDV requires cleavage of F protein precursor F0 (553 amino acid) into two subunits F1 and F2 [7]. The cleavage site amino acid sequence determines cleavage specificity and varies with the type of strain [8], [9]. The F protein cleavage site of less virulent strains of NDV consists of monobasic or dibasic amino acid residues [9]. The F protein cleavability by intracellular proteases do not take place due to the presence of one or two basic amino acids thus, extracellular proteases are required to cleave F protein limiting the tropism of NDV to respiratory and enteric tracts. In most cells, the polybasic amino acids of F protein in velogenic strains act as the cleavage recognition site for furin like proteases [8], [9].

It has been observed that F proteins of virulent NDV strains contain lysine (K) and arginine (R) at their cleavage site (^112^R-R-Q-R/K-R^116^), and a phenylalanine at position 117 of F_1_. This site is recognized by intracellular proteases, furin that cleave the polybasic cleavage site forming F1 subunit which is suggested to be the contributor of neurological effects [9], [10], [11]. It was postulated that the proper binding of furin protease, assisted by the presence of basic amino acids at the F protein cleavage site, leads to cleavage thus altering host-cell enzyme activity [11]. The variation in intracellular cleavage of virulent NDV F protein is observed to be dictated by the presence of arginine at position 113, 115 and 116 [12]. The substitution of the neutral amino acid, glutamine present at 114 position with an acidic or basic amino acid would attenuate NDV [4]. Furthermore, the attenuation of NDV by substituting valine to isoleucine at position 118 around the fusion cleavage site was also reported [4]. In another study, it has been shown that mutation at glycosylation site of F protein may enhance the virulence and pathogenicity of NDV [13]. It is also evident that mutation in the cytoplasmic domain of F protein can lead to the production of a hyperfusogenic virus that could ensure increased viral replication and pathogenesis in chickens [14]. Subtilisin like mammalian proteases, e.g., PC6 and PACE4 are reported as candidates for the cleavage of the F protein [15]. The F protein mediates virus penetration by inducing fusion between the viral envelope and host cell plasma membrane [3], [5], [12], [16], [17]. Various other factors are also accountable for the virulence of NDV [12]. In chicken and infected macrophages, F protein is a determinant of NDV virulence [6], [18]. We have shown that gradients of NDV virulence are multigenic, F protein is a major player of NDV virulence and pathogenicity and, the superiority of F as an antigen over HN for better and sterile immunity against NDV infection [19]. Based on our current understanding F glycoprotein is the most suitable protein for investigating the infectious capability of NDV strains. Based on pathogenic studies NDV is categorized into three major pathotypes: lentogenic (low virulence), mesogenic (moderate virulence) and velogenic (highly virulent) [20].

The synonymous codon usage is the non-random selection of frequently used codons, the selection of which is limited by codon bias for different genes [21], [22], [23], [24]. Synonymous codons are not used randomly as some codons are used more frequently than others [25]. Factors that may dictate synonymous codon usage bias include natural selection, mutational pressure, translational efficiency and compositional constraints of the mammalian genome [26], [27], [28]. Many studies have shown the contribution of codon usage bias patterns in order to understand the virus evolution [27], [29]. Although the factors responsible for the pathogenicity of the NDV due to F glycoprotein have been studied but the non-random synonymous codon usage variation in NDV isolates from different species has not been reported. A comprehensive analysis of the codon usage bias patterns of NDV isolates from different species may be necessary to understand the codon usage patterns in the virus evolution while crossing the species barrier. This analysis may pave way for future understanding of selection pressure due to host metabolome interaction and enable deciphering of the virus evolutionary trend among different species.

## Materials and Methods

### Gene sequences

Two hundred and one complete F gene sequences for NDV isolates from four major host species were obtained from the GenBank (Table 1). The four major avian species (chicken, duck, pigeon, and goose) were selected based on the availability of more than ten complete open reading frames (ORF) of the NDV F gene sequences from each in GenBank. The strains of NDV collected for the analysis were from all three major pathotypes, namely lentogenic, mesogenic, and velogenic. The codon usage pattern analysis was performed for the coding sequence of the F gene.

**Table 1:** List of Newcastle disease virus F gene used for analysis of synonymous codon usage in the study.

S.no	Accession Number	Cleavage Site amino acids 111 to 117	Pathotype	Species	GC3s (%)	GC (%)	N_c_	Mononucleotide frequencies (%)			
								A	C	G	T
1	JX524203	-G-G-K-Q-G-R*L-	Lentogenic	Chicken	44.4	45.3	57.8	29.30	22.98	22.32	25.39
2	HM117720	-G-R-R-Q-K-R*F-	Velogenic	Chicken	45.5	45.8	59.1	29.24	23.95	21.84	24.97
3	JX390609	-G-R-R-R-K-R*F-	Lentogenic	Chicken	44.4	45.1	58.1	29.84	23.41	21.66	25.09
4	FJ939313	-G-R-R-Q-K-R*F-	Velogenic	Chicken	43.6	44.5	59.6	30.69	23.53	21.00	24.79
5	GU187941	-G-R-R-Q-R-R*F-	Mesogenic	Chicken	42.0	45.2	55.0	29.72	23.47	21.72	25.09
6	FJ436305	-G-R-R-Q-R-R*F-	Mesogenic	Chicken	42.1	44.3	55.2	29.36	22.20	22.08	26.35
7	FJ436304	-G-R-R-Q-R-R*F-	Mesogenic	Chicken	41.9	44.4	55.2	29.24	22.20	22.20	26.35
8	FJ436303	-G-R-R-Q-R-R*F-	Mesogenic	Chicken	42.1	44.4	55.2	29.30	22.26	22.14	26.29
9	FJ436302	-G-R-R-Q-R-R*F-	Mesogenic	Chicken	41.9	44.4	55.1	29.30	22.20	22.14	26.35
10	HM357251	-G-G-R-Q-G-R*L-	Lentogenic	Chicken	43.0	44.5	59.4	30.26	23.23	21.30	25.21
11	KJ123642	-G-R-R-Q-K-R*F-	Velogenic	Chicken	43.4	44.9	59.5	29.82	23.11	21.75	25.32
12	FJ754271	-G-R-R-Q-K-R*F-	Velogenic	Chicken	40.7	44.0	54.9	30.02	22.80	21.18	25.99
13	JX110635	-G-G-K-Q-R-R*L-	Lentogenic	Chicken	44.2	45.1	57.9	29.36	22.86	22.26	25.51
14	KC461214	-G-R-R-Q-K-R*F-	Velogenic	Chicken	40.3	44.4	55.1	29.66	22.62	21.72	25.99
15	JX974435	-G-R-R-Q-K-R*F-	Velogenic	Chicken	45.3	45.7	58.0	29.18	23.77	21.90	25.15
16	FJ751919	-G-R-R-Q-K-R*F-	Velogenic	Chicken	44.9	44.8	57.0	29.48	23.16	21.66	25.69
17	FJ751918	-G-R-R-Q-K-R*F-	Velogenic	Chicken	44.9	44.8	56.9	29.36	23.16	21.66	25.81
18	JX316216	-G-R-R-Q-K-R*F-	Mesogenic	Chicken	42.9	45.3	58.5	29.48	23.16	22.08	25.27
19	JX867334	-G-R-R-Q-K-R*F-	Velogenic	Chicken	40.0	43.9	55.7	30.32	22.68	21.18	25.81
20	JX519467	-G-R-R-Q-K-R*F-	Velogenic	Chicken	39.9	44.2	55.3	29.90	22.62	21.54	25.93
21	JX119193	-R-R-R-Q-K-R*F-	Velogenic	Chicken	43.1	45.1	55.5	30.14	23.59	21.42	24.85
22	JN618349	-G-R-R-Q-K-R*F-	Velogenic	Chicken	41.5	44.4	54.6	29.96	23.10	21.24	25.69
23	JN618348	-G-R-R-Q-K-R*F-	Velogenic	Chicken	43.0	45.1	55.7	29.48	23.29	21.78	25.45
24	HQ697255	-G-R-R-R-K-R*F-	Lentogenic	Chicken	43.3	45.0	55.5	29.30	23.16	21.78	25.75
25	HQ697254	-G-R-R-Q-K-R*F-	Velogenic	Chicken	44.1	45.1	57.5	29.48	23.53	21.60	25.39
26	JN682211	-G-R-R-Q-K-R*F-	Velogenic	Chicken	39.5	43.9	53.7	30.20	22.56	21.30	25.93
27	JN688863	-G-E-Q-Q-E-R*L-	Lentogenic	Chicken	47.6	45.9	59.7	30.99	22.98	22.86	23.16
28	JN688862	-G-E-Q-Q-E-R*L-	Lentogenic	Chicken	47.3	45.7	56.4	31.05	22.74	22.86	23.35
29	JN800306	-G-R-R-Q-K-R*F-	Velogenic	Chicken	44.5	45.0	55.9	30.08	23.89	21.06	24.97
30	GU585905	-G-R-R-Q-R-R*F-	Mesogenic	Chicken	39.2	43.9	53.8	30.26	22.86	21.06	25.81
31	GU978777	-G-R-R-Q-K-R*F-	Velogenic	Chicken	43.8	45.0	58.3	30.32	23.53	21.42	24.73
32	AB853928	-G-R-R-Q-K-R*F-	Velogenic	Chicken	41.4	44.3	55.6	30.20	23.04	21.18	25.57
33	AB853927	-G-R-R-Q-K-R*F-	Velogenic	Chicken	41.9	44.7	54.4	29.54	23.16	21.54	25.75
34	AB853926	-G-R-R-Q-K-R*F-	Velogenic	Chicken	43.0	44.9	56.0	29.96	23.47	21.42	25.15
35	JX012096	-G-R-R-Q-K-R*F-	Velogenic	Chicken	44.3	45.3	57.2	29.90	23.77	21.54	24.79
36	JX193076	-G-R-R-Q-K-R*F-	Velogenic	Chicken	40.5	44.3	55.2	29.84	22.86	21.42	25.87
37	JX193075	-R-R-R-Q-K-R*F-	Velogenic	Chicken	42.0	44.6	54.4	29.54	22.86	21.72	25.87
38	JQ015297	-G-R-R-Q-K-R*F-	Velogenic	Chicken	41.5	44.7	55.5	30.02	23.41	21.30	25.27
39	JQ015296	-G-R-R-Q-K-R*F-	Velogenic	Chicken	42.0	44.9	55.0	30.02	23.59	21.30	25.09
40	JQ015295	-G-R-R-Q-K-R*F-	Velogenic	Chicken	42.0	45.0	55.1	30.02	23.65	21.30	25.03
41	HQ839733	-G-R-R-Q-K-R*F-	Velogenic	Chicken	42.3	44.7	57.2	30.32	23.89	20.70	25.09
42	JF950509	-G-R-R-Q-R-R*F-	Mesogenic	Chicken	41.9	44.7	55.1	29.00	22.44	22.20	26.35
43	FJ766529	-G-R-R-Q-K-R*F-	Velogenic	Chicken	42.0	44.5	55.9	30.20	23.35	21.12	25.33
44	FJ430159	-G-R-R-Q-R-R*F-	Mesogenic	Chicken	41.9	44.5	54.8	29.06	22.38	22.14	26.41
45	DQ485231	-R-R-R-Q-K-R*F-	Velogenic	Chicken	42.2	44.4	55.2	30.08	23.10	21.30	25.51
46	DQ485230	-G-R-R-Q-K-R*F-	Velogenic	Chicken	42.2	44.7	55.7	29.84	23.10	21.54	25.51
47	DQ485229	-G-R-R-Q-K-R*F-	Velogenic	Chicken	41.7	44.8	55.3	29.42	22.86	21.96	25.75
48	AY562991	-G-G-K-Q-G-R*L-	Lentogenic	Chicken	46.8	46.6	59.0	28.40	23.89	22.68	25.03
49	AY562988	-G-R-R-Q-K-R*F-	Velogenic	Chicken	43.8	45.3	53.9	29.48	23.47	21.78	25.27
50	AY562987	-G-R-R-Q-K-R*F-	Velogenic	Chicken	43.9	45.3	57.1	29.48	23.47	21.78	25.27
51	EF201805	-G-R-R-Q-R-R*F-	Mesogenic	Chicken	41.9	44.6	54.9	29.00	22.38	22.20	26.41
52	KJ019841	-G-G-R-Q-G-R*L-	Lentogenic	Chicken	43.0	44.4	58.9	30.45	23.16	21.24	25.15
53	KF935230	-G-R-R-Q-K-R*F-	Velogenic	Chicken	42.4	45.1	55.2	29.90	23.71	21.42	24.97
54	KF727980	-G-R-R-Q-K-R*F-	Velogenic	Chicken	41.2	45.0	56.1	29.66	23.35	21.66	25.33
55	JX443519	-G-G-K-Q-G-R*L-	Lentogenic	Chicken	44.4	45.3	57.7	29.30	22.98	22.32	25.39
56	JF966387	-G-R-R-Q-K-R*F-	Velogenic	Chicken	45.2	44.9	54.0	30.08	23.53	21.36	25.03
57	JF966386	-G-R-R-R-K-R*F-	Lentogenic	Chicken	43.1	45.5	53.0	30.14	23.95	21.48	24.43
58	JF966385	-G-R-R-Q-K-R*F-	Velogenic	Chicken	41.7	44.5	56.6	30.02	22.80	21.72	25.45
59	KC844235	-G-G-R-Q-G-R*L-	Lentogenic	Chicken	42.9	44.4	59.0	30.39	23.16	21.24	25.21
60	KC542914	-G-R-R-Q-K-R*F-	Velogenic	Chicken	40.9	44.5	53.8	29.96	23.16	21.30	25.57
61	KC542913	-G-R-R-Q-K-R*F-	Velogenic	Chicken	42.0	44.8	55.0	30.02	23.53	21.30	25.15
62	KC542912	-G-R-R-Q-K-R*F-	Velogenic	Chicken	42.0	44.8	55.0	30.02	23.53	21.30	25.15
63	KC542911	-G-R-R-Q-K-R*F-	Velogenic	Chicken	42.4	44.5	55.2	30.02	23.23	21.30	25.45
64	KC542910	-G-R-R-Q-K-R*F-	Velogenic	Chicken	42.8	44.8	58.0	29.96	23.47	21.30	25.27
65	KC542909	-G-R-R-Q-K-R*F-	Velogenic	Chicken	41.0	44.4	54.2	30.08	23.10	21.24	25.57
66	KC542908	-G-R-R-Q-K-R*F-	Velogenic	Chicken	40.9	44.1	54.6	30.08	22.92	21.12	25.87
67	KC542907	-G-R-R-Q-K-R*F-	Velogenic	Chicken	42.4	44.7	54.9	30.02	23.47	21.24	25.27
68	KC542906	-G-R-R-Q-K-R*F-	Velogenic	Chicken	42.1	44.7	55.0	30.02	23.47	21.24	25.27
69	KC542905	-G-R-R-Q-K-R*F-	Velogenic	Chicken	42.2	44.9	54.8	30.02	23.59	21.30	25.09
70	KC542904	-G-R-R-Q-K-R*F-	Velogenic	Chicken	41.7	44.7	54.4	29.90	23.29	21.36	25.45
71	KC542903	-G-R-R-Q-K-R*F-	Velogenic	Chicken	42.0	44.8	54.4	29.90	23.41	21.36	25.33
72	KC542902	-G-R-R-Q-K-R*F-	Velogenic	Chicken	42.5	44.7	55.0	29.72	23.04	21.60	25.63
73	KC542901	-G-R-R-Q-K-R*F-	Velogenic	Chicken	40.0	43.8	55.4	30.39	22.68	21.12	25.81
74	KC542900	-G-R-R-Q-K-R*F-	Velogenic	Chicken	40.0	43.8	55.4	30.39	22.68	21.12	25.81
75	KC542899	-G-R-R-Q-K-R*F-	Velogenic	Chicken	41.9	44.7	54.5	29.96	23.41	21.24	25.39
76	KC542898	-G-R-R-Q-K-R*F-	Velogenic	Chicken	41.3	44.4	55.9	29.90	22.92	21.48	25.69
77	KC542897	-G-R-R-Q-K-R*F-	Velogenic	Chicken	41.5	44.5	56.3	29.60	22.80	21.72	25.87
78	KC542896	-G-R-R-Q-K-R*F-	Velogenic	Chicken	41.5	44.5	54.3	30.08	23.29	21.18	25.45
79	KC542895	-G-R-R-Q-K-R*F-	Velogenic	Chicken	41.7	44.7	56.5	29.78	23.10	21.60	25.51
80	KC542894	-G-R-R-Q-K-R*F-	Velogenic	Chicken	40.9	44.5	53.8	29.96	23.16	21.30	25.57
81	KC542893	-G-R-R-Q-K-R*F-	Velogenic	Chicken	41.1	44.5	54.4	29.96	23.16	21.30	25.57
82	KC542892	-G-R-R-Q-K-R*F-	Velogenic	Chicken	41.5	44.6	54.8	29.96	23.29	21.30	25.45
83	JN682210	-G-R-R-Q-K-R*F-	Velogenic	Chicken	39.5	43.9	53.7	30.14	22.62	21.30	25.93
84	**JN986837**	-G-R-R-Q-K-R*F-	**Velogenic**	**Chicken**	44.1	45.2	**52.3**	29.90	23.83	21.36	24.91
85	JN986838	-G-R-R-Q-K-R*F-	Velogenic	Chicken	42.4	44.7	55.8	30.02	23.23	21.48	25.27
86	JQ247691	-G-R-R-Q-K-R*F-	Velogenic	Chicken	44.8	45.6	59.6	29.42	23.71	21.84	25.04
87	JN400897	-G-R-R-Q-K-R*F-	Velogenic	Chicken	41.9	44.6	54.5	29.96	23.29	21.30	25.45
88	JN400896	-G-R-R-Q-K-R*F-	Velogenic	Chicken	42.0	44.8	55.9	29.60	23.10	21.72	25.57
89	JF343539	-G-R-R-Q-K-R*F-	Velogenic	Chicken	42.2	44.7	55.7	29.84	23.10	21.54	25.51
90	JF950510	-G-G-R-Q-G-R*L-	Lentogenic	Chicken	43.0	44.5	59.1	30.39	23.23	21.24	25.15
91	GU564399	-R-R-R-Q-K-R*F-	Velogenic	Chicken	42.2	44.6	55.8	29.84	23.10	21.48	25.57
92	HQ266603	-G-R-R-R-R-R*F-	Lentogenic	Chicken	47.2	46.5	58.9	28.46	23.53	22.98	25.03
93	HQ266602	-G-R-R-R-R-R*F-	Lentogenic	Chicken	46.4	46.5	57.8	28.46	23.35	23.10	25.09
94	GQ994434	-G-G-K-Q-G-R*L-	Lentogenic	Chicken	41.4	44.6	56.6	29.72	22.98	21.54	25.69
95	GQ994433	-G-G-R-Q-G-R*L-	Lentogenic	Chicken	41.9	44.9	54.8	29.42	22.80	21.96	25.75
96	FJ386396	-G-R-R-Q-K-R*F-	Velogenic	Chicken	44.1	44.9	58.6	29.96	23.59	21.30	25.15
97	FJ386395	-G-R-R-Q-K-R*F-	Velogenic	Chicken	44.3	44.9	59.6	30.39	23.47	21.42	24.73
98	FJ386394	-G-R-R-Q-K-R*F-	Velogenic	Chicken	44.3	44.9	58.9	30.39	23.41	21.48	24.73
99	FJ386393	-G-R-R-Q-K-R*F-	Velogenic	Chicken	44.3	44.9	58.6	30.39	23.77	21.12	24.73
100	FJ386392	-G-R-R-Q-K-R*F-	Velogenic	Chicken	44.3	44.9	59.6	30.45	23.29	21.60	24.67
101	AB605247	-G-R-R-Q-K-R*F-	Velogenic	Chicken	44.5	45.3	55.0	28.76	23.35	21.96	25.93
102	FJ217666	-G-R-R-Q-K-R*F-	Velogenic	Chicken	43.3	44.9	56.3	29.36	22.98	21.96	25.69
103	FJ217665	-G-R-R-Q-K-R*F-	Velogenic	Chicken	43.3	44.9	58.7	29.42	23.04	21.84	25.69
104	JF966389	-G-R-R-Q-K-R*F-	Velogenic	Chicken	46.2	45.0	56.8	30.20	23.59	21.42	24.79
105	JF966388	-G-R-R-Q-K-R*F-	Velogenic	Chicken	45.8	44.7	54.0	30.32	23.35	21.36	24.97
106	KC205479	-G-R-R-H-K-R*F-	NA	Chicken	44.4	45.2	58.5	30.45	24.07	21.12	24.37
107	KC205478	-G-R-R-Q-K-R*F-	Velogenic	Chicken	45.1	45.7	57.3	30.08	24.19	21.48	24.25
108	KC205475	-G-R-R-Q-K-R*F-	Velogenic	Chicken	45.3	45.5	58.9	30.02	24.13	21.42	24.43
109	GU166154	-R-R-R-Q-K-R*F-	Velogenic	Chicken	42.4	44.2	55.2	29.66	22.50	21.72	26.11
110	KC205477	-G-R-R-Q-K-R*F-	Velogenic	Chicken	44.0	45.2	58.2	30.39	24.07	21.18	24.37
111	**KC205476**	-G-R-R-R-K-R*F-	**Lentogenic**	**Chicken**	45.5	45.5	**60.0**	30.14	23.89	21.66	24.31
112	EF520718	-G-R-R-Q-K-R*F-	Velogenic	Chicken	45.3	45.3	57.9	29.96	24.13	21.18	24.73
113	FJ436306	-G-R-R-Q-R-R*F-	Mesogenic	Duck	41.8	44.4	55.0	29.30	22.26	22.14	26.29
114	HM125898	-G-G-K-Q-G-R*L-	Lentogenic	Duck	47.9	46.8	58.9	28.52	23.89	22.92	24.67
115	KF771883	-G-R-R-Q-K-R*F-	Velogenic	Duck	42.2	44.5	55.7	30.02	23.35	21.18	25.45
116	KF361507	-G-E-R-Q-E-R*L-	Lentogenic	Duck	50.2	47.0	56.1	30.20	23.71	23.16	22.92
117	FJ754272	-G-R-R-Q-K-R*F-	Velogenic	Duck	41.7	44.7	55.2	29.48	22.74	21.96	25.81
118	JX401405	-G-G-K-Q-G-R*L-	Lentogenic	Duck	48.1	46.9	57.7	28.58	24.07	22.80	24.55
119	JX401404	-G-G-K-Q-G-R*L-	Lentogenic	Duck	46.9	46.5	58.9	28.88	24.01	22.50	24.61
120	JX401403	-G-G-K-Q-G-R*L-	Lentogenic	Duck	46.4	46.3	58.7	28.76	23.65	22.62	24.97
121	JN688864	-G-E-Q-Q-E-R*L-	Lentogenic	Duck	47.5	45.6	57.3	31.11	22.62	22.86	23.41
122	FJ794269	-G-E-Q-Q-E-R*L-	Lentogenic	Duck	48.1	46.1	56.2	30.87	22.86	23.10	23.16
123	GQ849007	-G-R-R-Q-K-R*F-	Velogenic	Duck	42.0	45.1	54.7	29.48	23.29	21.84	25.39
124	KC894391	-G-G-K-Q-G-R*L-	Lentogenic	Duck	48.1	47.1	58.6	28.34	23.95	23.10	24.61
125	JX193083	-G-G-K-Q-G-R*L-	Lentogenic	Duck	46.1	46.2	58.4	28.76	23.65	22.50	25.09
126	JX193082	-G-G-R-Q-G-R*L-	Lentogenic	Duck	42.8	44.4	58.6	30.32	23.16	21.24	25.27
127	JX193081	-G-G-K-Q-G-R*L-	Lentogenic	Duck	46.5	46.7	58.3	28.58	23.95	22.74	24.73
128	JX193080	-G-G-R-Q-G-R*L-	Lentogenic	Duck	42.9	44.5	58.5	30.26	23.16	21.30	25.27
129	JX193079	-G-G-K-Q-G-R*L-	Lentogenic	Duck	44.3	45.3	58.0	29.18	22.98	22.26	25.57
130	JX193078	-G-G-K-Q-G-R*L-	Lentogenic	Duck	45.9	46.2	58.9	28.88	23.71	22.44	24.97
131	**JX193077**	-G-G-K-Q-G-R*L-	**Lentogenic**	**Duck**	46.9	46.3	**59.3**	28.88	23.59	22.68	24.85
132	HQ008337	-G-E-R-Q-E-R*L-	Lentogenic	Duck	49.1	47.0	54.9	30.26	23.77	23.16	22.80
133	GQ288392	-G-E-K-Q-G-R*L-	Lentogenic	Duck	42.7	45.0	58.2	29.36	23.10	21.84	25.69
134	GQ288391	-G-G-K-Q-G-R*L-	Lentogenic	Duck	43.0	45.0	57.0	29.42	23.04	21.96	25.57
135	GQ288390	-G-E-K-Q-G-R*L-	Lentogenic	Duck	43.4	45.1	57.6	29.18	23.10	22.02	25.69
136	GQ288389	-G-E-K-Q-G-R*L-	Lentogenic	Duck	43.0	45.1	58.1	29.30	23.23	21.84	25.63
137	GQ288380	-G-E-K-Q-G-R*L-	Lentogenic	Duck	43.0	44.9	58.8	29.42	23.16	21.72	25.69
138	GQ288379	-G-E-K-Q-G-R*L-	Lentogenic	Duck	43.0	45.1	58.2	29.24	23.16	21.90	25.69
139	GQ288377	-G-E-K-Q-G-R*L-	Lentogenic	Duck	43.4	45.3	57.5	29.06	23.23	22.08	25.63
140	DQ097393	-G-G-R-Q-G-R*L-	Lentogenic	Duck	50.4	47.6	55.8	29.90	23.89	23.59	22.62
141	KC920893	-G-R-R-Q-R-R*F-	Velogenic	Duck	41.9	44.4	55.1	29.24	22.20	22.20	26.35
142	JN400895	-G-R-R-Q-K-R*F-	Velogenic	Duck	41.8	45.0	54.8	29.84	23.59	21.42	25.15
143	HQ717357	-G-R-R-Q-K-R*F-	Velogenic	Duck	40.3	44.3	55.6	30.02	23.10	21.18	25.69
144	HQ317395	-G-R-R-Q-K-R*F-	Velogenic	Duck	41.9	45.0	54.8	29.78	23.59	21.42	25.21
145	HQ317394	-G-R-R-Q-R-R*F-	Mesogenic	Duck	41.9	44.4	55.1	29.24	22.20	22.14	26.41
146	HM063422	-G-E-R-Q-G-R*L-	Lentogenic	Duck	46.1	46.2	58.8	28.94	23.89	22.32	24.85
147	JF893453	-G-E-R-Q-E-R*L-	Lentogenic	Duck	49.5	47.0	57.1	30.08	23.59	23.35	22.98
148	HQ412767	-G-E-R-Q-G-R*L-	Lentogenic	Duck	48.9	46.8	55.8	30.45	23.71	23.04	22.8
149	**HM188399**	-G-R-R-Q-K-R*F-	**Velogenic**	**Duck**	41.7	44.8	**54.4**	29.84	23.41	21.36	25.39
150	EF521889	-G-R-R-Q-K-R*F-	Velogenic	Duck	44.4	44.8	55.4	29.48	23.23	21.60	25.69
151	FJ410147	-G-R-R-Q-K-R*F-	Velogenic	Pigeon	43.8	45.4	57.8	30.39	24.31	21.12	24.19
152	FJ410145	-G-R-R-Q-K-R*F-	Velogenic	Pigeon	43.6	45.3	57.9	30.51	24.25	21.06	24.19
153	FJ766531	-G-R-R-R-K-R*F-	Lentogenic	Pigeon	45.6	45.8	56.9	30.20	24.37	21.42	24.01
154	FJ766530	-G-R-R-R-K-R*F-	Lentogenic	Pigeon	45.5	45.8	56.7	30.20	24.37	21.42	24.01
155	FJ766528	-E-K-R-Q-K-R*F-	Velogenic	Pigeon	44.3	45.2	56.4	30.45	24.13	21.06	24.37
156	FJ766527	-G-R-R-R-K-R*F-	Lentogenic	Pigeon	45.2	45.7	56.1	30.26	24.31	21.36	24.07
157	FJ766526	-G-R-R-Q-K-R*F-	Velogenic	Pigeon	47.0	46.1	57.3	30.20	24.85	21.24	23.71
158	KC013040	-E-R-R-Q-K-R*F-	Velogenic	Pigeon	42.4	44.6	54.6	30.87	23.83	20.76	24.55
159	KC013039	-V-R-R-K-K-R*F-	Velogenic	Pigeon	45.1	45.3	58.3	30.14	23.83	21.48	24.55
160	KC013038	-V-R-R-K-K-R*F-	Velogenic	Pigeon	44.1	45.1	58.0	30.20	23.77	21.30	24.73
161	KC013037	-V-R-R-K-K-R*F-	Velogenic	Pigeon	44.0	45.1	57.4	29.90	23.71	21.36	25.03
162	KC013036	-V-R-R-K-K-R*F-	Velogenic	Pigeon	44.2	44.9	58.1	30.32	23.77	21.12	24.79
163	KC013035	-V-R-R-K-K-R*F-	Velogenic	Pigeon	43.7	44.7	57.6	30.39	23.59	21.06	24.97
164	KC013034	-V-R-R-K-K-R*F-	Velogenic	Pigeon	43.8	45.0	57.9	30.20	23.83	21.18	24.79
165	KC013033	-V-R-R-K-K-R*F-	Velogenic	Pigeon	44.2	45.1	57.9	30.20	23.83	21.30	24.67
166	KC013032	-V-R-R-K-K-R*F-	Velogenic	Pigeon	44.6	45.1	57.9	30.14	23.77	21.30	24.79
167	KC013031	-E-R-R-Q-K-R*F-	Velogenic	Pigeon	44.4	44.8	56.8	30.81	24.01	20.76	24.43
168	JX486557	-R-R-R-Q-K-R*F-	Velogenic	Pigeon	46.6	45.9	56.9	30.26	24.85	21.00	23.89
169	JX486556	-G-R-R-Q-K-R*F-	Velogenic	Pigeon	46.2	46.2	57.8	30.08	24.79	21.36	23.77
170	JX486555	-G-R-R-Q-K-R*F-	Velogenic	Pigeon	46.2	46.2	57.8	30.08	24.79	21.36	23.77
171	JX486554	-G-R-R-Q-K-R*F-	Velogenic	Pigeon	46.4	46.1	56.8	30.32	24.97	21.06	23.65
172	JX486553	-G-R-R-Q-K-R*F-	Velogenic	Pigeon	46.6	46.2	57.1	30.2	24.97	21.18	23.65
173	JX486552	-G-R-R-Q-K-R*F-	Velogenic	Pigeon	44.1	45.1	57.4	30.75	24.31	20.82	24.13
174	JX486551	-G-R-R-Q-K-R*F-	Velogenic	Pigeon	44.7	45.9	54.2	29.96	24.25	21.60	24.19
175	JX486550	-G-R-R-Q-K-R*F-	Velogenic	Pigeon	45.7	45.6	56.2	30.57	24.67	20.88	23.89
176	JF827026	-G-K-R-Q-K-R*F-	Velogenic	Pigeon	43.8	45.0	58.2	30.32	23.95	21.06	24.67
177	JX901110	-G-R-R-Q-K-R*F-	Velogenic	Pigeon	45.8	45.8	54.4	30.26	24.49	21.24	24.01
178	JX901109	-G-R-R-Q-K-R*F-	Velogenic	Pigeon	46.0	45.8	54.4	30.26	24.55	21.24	23.95
179	GQ429292	-V-R-R-K-K-R*F-	Velogenic	Pigeon	46.0	45.8	58.6	30.08	24.43	21.30	24.19
180	JQ993431	-G-R-R-Q-K-R*F-	Velogenic	Pigeon	44.8	45.3	57.7	30.75	24.49	20.82	23.95
181	JQ979176	-G-R-R-Q-K-R*F-	Velogenic	Pigeon	45.1	45.5	57.4	30.69	24.61	20.88	23.83
182	**JF827027**	-G-K-R-Q-K-R*F-	**Velogenic**	**Pigeon**	43.6	45.1	**60.5**	30.08	23.77	21.30	24.85
183	JN986839	-G-R-R-Q-K-R*F-	Velogenic	Pigeon	46.2	46.0	56.2	30.20	24.67	21.30	23.83
184	**FJ986192**	-G-R-R-Q-R-R*L-	**Lentogenic**	**Pigeon**	42.0	45.3	**53.8**	29.66	23.53	21.72	25.09
185	HM063425	-G-R-R-Q-K-R*F-	Velogenic	Pigeon	46.2	45.9	56.5	30.14	24.43	21.48	23.95
186	EF026583	-G-R-R-Q-K-R*F-	Velogenic	Pigeon	45.8	45.8	54.4	30.26	24.49	21.24	24.01
187	EF026579	-G-R-R-Q-K-R*F-	Velogenic	Pigeon	45.8	45.8	54.5	30.26	24.55	21.24	23.95
188	AJ880277	-G-G-R-Q-K-R*F-	Velogenic	Pigeon	44.2	45.6	58.0	30.39	24.43	21.12	24.07
189	DQ417113	-E-K-R-Q-K-R*F-	Mesogenic	Pigeon	45.8	45.1	56.5	30.32	23.95	21.18	24.55
190	JF713701	-G-R-R-Q-K-R*F-	Velogenic	Pigeon	48.0	46.3	54.8	29.96	24.91	21.42	23.71
191	FJ754273	-G-R-R-Q-K-R*F-	Velogenic	Goose	41.9	44.8	56.1	29.48	22.92	21.84	25.75
192	KC551967	-G-R-R-Q-K-R*F-	Velogenic	Goose	44.3	45.3	53.5	30.45	24.29	21.06	24.31
193	KC152049	-G-R-R-Q-K-R*F-	Velogenic	Goose	44.3	45.3	53.4	30.39	24.19	21.12	24.31
194	KC152048	-G-R-R-Q-K-R*F-	Velogenic	Goose	44.1	45.3	53.6	30.32	24.01	21.30	24.37
195	**JN688865**	-G-E-Q-Q-G-R*L-	**Lentogenic**	**Goose**	48.6	46.1	**53.1**	30.32	22.38	23.65	23.65
196	JN631747	-G-R-R-Q-K-R*F-	Velogenic	Goose	41.7	44.6	54.9	30.02	23.29	21.30	25.39
197	GU143550	-G-R-R-Q-K-R*F-	Velogenic	Goose	41.1	44.5	55.3	29.54	22.74	21.72	25.99
198	DQ659677	-G-R-R-Q-K-R*F-	Velogenic	Goose	41.7	44.7	54.6	29.66	22.98	21.60	25.75
199	FJ430160	-G-R-R-Q-R-R*F-	Mesogenic	Goose	42.1	44.6	55.1	29.00	22.38	22.20	26.41
200	**AB524405**	-G-E-R-Q-E-R*L-	**Lentogenic**	**Goose**	49.6	47.1	**56.8**	29.96	23.47	23.53	23.04
201	JF340367	-G-R-R-Q-K-R*F-	Velogenic	Goose	41.9	44.8	55.7	29.72	23.23	21.60	25.45

^*^Represents cleavage point, NA represents an unknown pathotype. The pathotype has been identified based on the cleavage site. Bold letters indicates the lower and higher value of Nc.

### Codon usage analysis

The patterns of codon usage were analyzed for the two hundred and one F gene sequences for NDV isolated from four major host species. The relative synonymous codon usage (RSCU) values of each codon for the F gene were calculated using Codon W 1.4.4 software. The calculation of RSCU index enables the characterization of synonymous usage of codons and is expressed as the ratio of the observed usage of codons to the expected value if all codons were used frequently. The RSCU value of 1 indicates that the codon is chosen randomly and evenly, RSCU >1 indicates that the codon usage is more frequent than the expected, and RSCU <1 indicates that the codon chosen is less frequent [30].

RSCU calculation formulae: RSCU  =  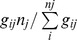


Where g_ij_  =  observed number of codons for i^th^ codon for j^th^ amino acid that has n_j_ kinds of synonymous codon.

#### Effective number of codons

Quantification of the codon usage bias of the ORF in a gene is calculated by the effective number of codons (ENC or Nc). The Nc best estimates the absolute synonymous codon usage bias in a gene.

ENC calculation formulae: ENC  =  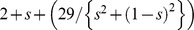


where *s* represents the value of G+C at the third codon position (GC3) [31]. Nc has been calculated using the Codon W 1.4.4 program. The Nc value was correlated to the percentage of GC3s. In case of biased codon usage only one codon for each amino acid is used and the Nc equals to 20. In case the Nc value is 61 then there is no bias in codon usage and all synonymous codons are equally used.

#### Codon adaptation index (CAI)

The CAI requires a reference set of highly expressed known gene and enables the estimation of the amount of bias (Codon W 1.4.4 program). The high value of CAI refers higher codon usage bias and expression level [32], [33], [34]. The CAI index is defined as the geometric mean of relative adaptiveness values. Non-synonymous codons and termination codons (dependent on genetic code) are excluded from the calculation. CAI values range from 0 to 1, with higher values indicating a higher proportion of the most abundant codons [35].

### Chemical property analysis of amino acids using various indices

#### Aliphatic Index

The aliphatic index (AI) refers to the relative volume of a protein that is occupied by aliphatic side chains (alanine, isoleucine, leucine and valine) and contributes to the increased thermo-stability observed for globular proteins. The AI of a protein is calculated according to the following formula [36].

Aliphaatic index (AI)  = 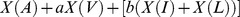


Here: X(A), X(V), X(I), and X(L) are mole percent (100 X mole fraction) of alanine, valine, isoleucine, and leucine, respectively. The coefficients a, b are the relative volumes of valine side chains (a  =  2.9) and of Leu/Ile side chains (b  =  3.9) relative to that of alanine side chains.

#### Grand average of hydropathy (GRAVY)

GRAVY is calculated as the arithmetic mean of the sum of the hydrophobic indices of each amino acid [37].

#### Correspondence analysis

Principal component analysis (PCA) was performed using the software XLSTAT version 2013.5.02. The PCA provides information regarding the major trend involved in the codon usage patterns measured from RSCU values and are calculated from 59 codons excluding methionine, tryptophan and all termination codons [38]. Correlation analysis was performed for the first two axes of PCA (PC1 and PC2). Pearson rank correlation analysis was performed to infer the relationships between the two axes of PCA and different variables like GRAVY, aromaticity index, aliphaticity index and GC3s.

### Phylogenetic analysis

#### Major avian-host species of NDV

The phylogenetic relationship of the four major avian host species of NDV was studied using the MEGA6 software. The mitochondrial DNA is an important data source in building the phylogenetic [39]. Advantages of mitochondrial genome over nuclear gene is that they are unlikely to have undergone many intra specific recombination events [40]. In order to confer the phylogenetic relationship of the major avian host species the mitochondrial genome reference sequence for the four major avian host species *Anas platyrhynchos* (Duck, ref seq. NC009684, 16604bp), *Gallus gallus* (Chicken, ref seq. NC001323, 16775bp), *Anser anser* (Goose, ref seq. NC011196, 16738bp) and *Columba livia* (Pigeon, ref seq. NC013978, 17229bp) were downloaded from GenBank. The neighbor-joining method was used with parameters including pairwise deletion, 1000 replicates for bootstrap analysis and Dayhoff substitution model.

#### NDV strains

The phylogenetic relationship between the NDV strains was studied using MEGA6 software. The 201 complete F gene sequences were obtained from GenBank (Table 1). For the ease of understanding the labeling was done as accession no/virulence/host species. In the label L, M, V, CH, DK, GE and PN stand for lentogenic, mesogenic, velogenic, chicken, duck, goose and pigeon respectively. The neighbor-joining method was used with parameters including pairwise deletion, 1000 replicates for bootstrap analysis and Jukes-Cantor substitution model.

## Results

### Codon usage bias of fusion (F) gene in four major host species

**Figure 1:**
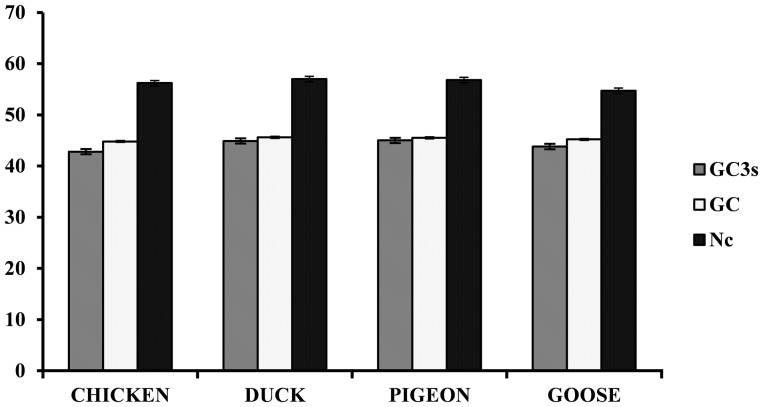
Improved effective number of codons index (Nc) as a measure of overall average codon usage bias (CUB) in four different species. The actual mean Nc, mean GC3s and mean GC calculated at 95% confidence.

The Nc value is the determinant of the degree of bias in codon usage. The four major host species showed a range of Nc values. For chicken, the maximum and minimum Nc values were 60 and 52.3, respectively. For duck, the maximum and minimum Nc values were 59.3 and 54.4, respectively. For pigeon, the maximum and minimum Nc values were 60.5 and 53.8, respectively. For goose, the maximum and minimum Nc values were 56.8 and 53.1, respectively (Table 1). The mean Nc was found to be maximum for duck and minimum for the goose. The GC3 is the amount of G + C at the third position whereas the GC is the total amount of G +C. The pigeons showed the maximum value of mean GC3s while the minimum value of mean GC3s was calculated for chicken. Similarly, maximum value of mean GC was calculated for a duck while chicken showed the minimum value (Figure 1). Slight variation in the values of mean GC3s, GC and Nc for duck and pigeon was observed. The mean GC was found to be greater than mean GC3s for all four species.

### Species specific identification of optimal codons

Analysis of codon based on RSCU values showed 21 optimal codons for 19 different amino acids, preferentially used for F gene of NDV (Table 2). A preferential usage of the optimal codons was compared against the species' specific codon usage and the frequencies of optimal codon usage (FOP) in all species were calculated [41]. Species specific codon usage was compared with the optimal codon usage. However, analysis within species showed maximum similarity with the optimal codon usage for chicken followed by goose, duck and minimum for pigeon isolate (Table 3).

**Table 2:** List of species specific synonymous codon usage in F gene of Newcastle disease virus. N is the number of codons; RSCU is cumulative relative synonymous codon usage.

Amino acid	Codon	Chicken		Duck		Pigeon		Goose		Overall	
		N	RSCU	N	RSCU	N	RSCU	N	RSCU	N	RSCU
Phe	UUU	**497**	**1.21**	**140**	**1.06**	**192**	**1.21**	**46**	**1.10**	**875**	**1.18**
	UUC	327	0.79	123	0.94	125	0.79	38	0.90	613	0.82
Leu	UUA	**1580**	**1.20**	436	0.98	**599**	**1.25**	**137**	**1.06**	**2752**	**1.16**
	UUG	1267	0.96	**467**	**1.05**	294	0.62	128	0.99	2156	0.91
Tyr	UAU	**1094**	**1.09**	**335**	**1.01**	300	0.88	**130**	**1.29**	**1859**	**1.04**
	UAC	921	0.91	326	0.99	**381**	**1.12**	71	0.71	1699	0.96
Stop	UAA	6	0.16	7	0.55	2	0.15	3	0.71	18	0.27
Stop	UAG	0	0.00	0	0.00	0	0.00	0	0.00	0	0.00
Leu	CUU	**1408**	**1.07**	451	1.01	522	1.09	124	0.96	2505	1.06
	CUC	1187	0.90	416	0.93	478	1.00	123	0.95	2204	0.93
	CUA	1123	0.85	372	0.84	372	0.78	121	0.94	1988	0.84
	CUG	1353	1.03	**529**	**1.19**	**602**	**1.26**	**141**	**1.09**	**2625**	**1.11**
His	CAU	**203**	**1.57**	**49**	**1.51**	**99**	**1.47**	**22**	**1.69**	**373**	**1.54**
	CAC	55	0.43	16	0.49	36	0.53	4	0.31	111	0.46
Gln	CAA	**1569**	**1.04**	**490**	**0.89**	**657**	**1.27**	145	0.96	**2861**	**1.05**
	CAG	1454	0.96	611	1.11	381	0.73	**156**	**1.04**	2602	0.95
Ile	AUU	1169	0.72	397	0.73	316	0.52	110	0.68	1992	0.68
	AUC	1570	0.97	552	1.01	632	1.03	170	1.06	2924	1.00
	AUA	**2101**	**1.30**	**683**	**1.26**	**886**	**1.45**	**203**	**1.26**	**3873**	**1.32**
Met	AUG	1361	1.00	492	1.00	443	1.00	132	1.00	2428	1.00
Asn	AAU	**2433**	**1.31**	**779**	**1.25**	**861**	**1.26**	**229**	**1.27**	**4302**	**1.28**
	AAC	1293	0.69	471	0.75	511	0.74	132	0.73	2407	0.72
Lys	AAA	1141	0.88	408	0.87	**509**	**1.09**	101	0.80	2159	0.91
	AAG	**1461**	**1.12**	**527**	**1.13**	427	0.91	**153**	**1.20**	**2568**	**1.09**
Val	GUU	807	0.76	273	0.75	204	0.57	71	0.67	1355	0.71
	GUC	**1427**	**1.34**	**429**	**1.18**	**526**	**1.46**	**138**	**1.30**	**2520**	**1.33**
	GUA	1097	1.03	385	1.06	353	0.98	121	1.14	1956	1.03
	GUG	937	0.88	364	1.00	361	1.00	94	0.89	1756	0.93
Asp	GAU	**1194**	**1.07**	**399**	**1.01**	**442**	**1.05**	109	0.99	**2144**	**1.05**
	GAC	1031	0.93	389	0.99	396	0.09	**112**	**1.01**	1928	0.95
Glu	GAA	**1211**	**1.20**	**408**	**1.13**	**376**	**1.09**	**118**	**1.17**	**2113**	**1.17**
	GAG	801	0.80	313	0.87	313	0.91	84	0.83	1511	0.83
Ser	UCU	1105	1.19	408	1.34	463	1.39	115	1.28	2091	1.26
	UCC	880	0.95	262	0.86	192	0.57	77	0.86	1411	0.85
	UCA	**1651**	**1.78**	**436**	**1.43**	**651**	**1.95**	**155**	**1.73**	**2893**	**1.75**
	UCG	379	0.41	208	0.68	124	0.37	36	0.40	747	0.45
Cys	UGU	**937**	**1.35**	**299**	**1.23**	233	0.92	**93**	**1.38**	**1562**	**1.24**
	UGC	455	0.65	189	0.77	**276**	**1.08**	42	0.62	962	0.76
Stop	UGA	**106**	**2.84**	**31**	**2.45**	**38**	**2.85**	**8**	**2.18**	**183**	**2.73**
Trp	UGG	119	1.00	39	1.00	42	1.00	11	1.00	211	1.00
Pro	CCU	**744**	**1.46**	**228**	**1.35**	174	0.96	**68**	**1.39**	**1214**	**1.34**
	CCC	454	0.89	193	1.14	**208**	**1.15**	51	1.04	906	1.00
	CCA	531	1.04	148	0.87	198	1.09	47	0.96	924	1.02
	CCG	308	0.60	108	0.64	146	0.80	30	0.61	592	0.65
Arg	CGU	**207**	**0.57**	**94**	**0.88**	43	0.34	16	0.45	**360**	**0.57**
	CGC	183	0.50	52	0.49	**85**	**0.67**	**22**	**0.62**	342	0.54
	CGA	120	0.33	18	0.17	80	0.63	10	0.28	228	0.36
	CGG	193	0.53	62	0.58	45	0.35	19	0.54	319	0.50
Thr	ACU	1936	1.30	641	1.23	615	1.16	165	1.12	3357	1.25
	ACC	1668	1.12	605	1.16	621	1.17	167	1.14	3061	1.14
	ACA	**1984**	**1.33**	**743**	**1.43**	**787**	**1.49**	**214**	**1.46**	**3728**	**1.39**
	ACG	387	0.26	91	0.18	96	0.18	42	0.286	616	0.23
Ser	AGU	492	0.53	191	0.63	181	0.54	56	0.63	920	0.56
	AGC	**1057**	**1.14**	**308**	**1.02**	**393**	**1.18**	**98**	**1.09**	**1856**	**1.12**
Arg	AGA	**871**	**2.39**	188	1.77	192	1.51	**79**	**2.23**	**1330**	**0.70**
	AGG	612	1.68	**224**	**2.11**	**319**	**2.51**	67	1.89	1222	0.64
Ala	GCU	1206	0.94	426	0.98	344	0.77	113	0.90	2089	0.91
	GCC	1293	1.00	457	1.06	532	1.18	123	0.98	2405	1.05
	GCA	**2101**	**1.63**	**714**	**1.65**	**675**	**1.51**	**220**	**1.75**	**3710**	**1.62**
	GCG	553	0.43	133	0.31	238	0.53	47	0.37	971	0.42
.Gly	GGU	**1211**	**1.34**	**427**	**1.12**	305	0.79	107	0.98	2050	1.03
	GGC	1178	1.30	322	0.84	**528**	**1.36**	114	1.04	2142	1.08
	GGA	891	0.99	328	0.86	262	0.68	97	0.89	1578	0.80
	GGG	1133	1.25	452	1.18	455	1.17	**119**	**1.09**	**2159**	**1.09**

The N and RSCU value of codons more preferentially used for each amino acid are displayed in bold. The overall values depict the codon usage for the F gene for all isolates.

**Table 3:** Species specific codon usage of F gene in Newcastle disease virus.

Amino acid	Codon	Codons generally used				Codons predominantly used in F gene
		Chicken	Duck	Pigeon	Goose	
Phe	UUU	**UUU**	**UUU**	**UUU**	**UUU**	**UUU**
	UUC					
Leu	UUA	**UUA**		**UUA**	**UUA**	**UUA**
	UUG		UUG			
Tyr	UAU	**UAU**	**UAU**		**UAU**	**UAU**
	UAC			UAC		
ter	UAA	–	–	–	–	–
ter	UAG	–	–	–	–	–
Leu	CUU	CUU				**CUG**
	CUC					
	CUA					
	CUG		**CUG**	**CUG**	**CUG**	
His	CAU	**CAU**	**CAU**	**CAU**	**CAU**	**CAU**
	CAC					
Gln	CAA	**CAA**	**CAA**	**CAA**		**CAA**
	CAG				CAG	
Ile	AUU					**AUA**
	AUC					
	AUA	**AUA**	**AUA**	**AUA**	**AUA**	
Met	AUG	**AUG**	**AUG**	**AUG**	**AUG**	**AUG**
Asn	AAU	**AAU**	**AAU**	**AAU**	**AAU**	**AAU**
	AAC					
Lys	AAA			AAA		**AAG**
	AAG	**AAG**	**AAG**		**AAG**	
Val	GUU					**GUC**
	GUC	**GUC**	**GUC**	**GUC**	**GUC**	
	GUA					
	GUG					
Asp	GAU	**GAU**	**GAU**	**GAU**		**GAU**
	GAC				GAC	
Glu	GAA	**GAA**	**GAA**	**GAA**	**GAA**	**GAA**
	GAG					
Ser	UCU					**UCA**
	UCC					
	UCA	**UCA**	**UCA**	**UCA**	**UCA**	
	UCG					
Cys	UGU	**UGU**	**UGU**		**UGU**	**UGU**
	UGC			UGC		
ter	UGA	**UGA**	**UGA**	**UGA**	**UGA**	**UGA**
Trp	UGG	**UGG**	**UGG**	**UGG**	**UGG**	**UGG**
Pro	CCU	**CCU**	**CCU**		**CCU**	**CCU**
	CCC			CCC		
	CCA					
	CCG					
Arg	CGU	**CGU**	**CGU**			**CGU**
	CGC			CGC	CGC	
	CGA					
	CGG					
Thr	ACU					**ACA**
	ACC					
	ACA	**ACA**	**ACA**	**ACA**	**ACA**	
	ACG					
Ser	AGU					**AGC**
	AGC	**AGC**	**AGC**	**AGC**	**AGC**	
Arg	AGA	**AGA**			**AGA**	**AGA**
	AGG		AGG	AGG		
Ala	GCU					**GCA**
	GCC					
	GCA	**GCA**	**GCA**	**GCA**	**GCA**	
	GCG					
Gly	GGU	GGU	GGU			**GGG**
	GGC					
	GGA					
	GGG			**GGG**	**GGG**	

### Nc plot

**Figure 2:**
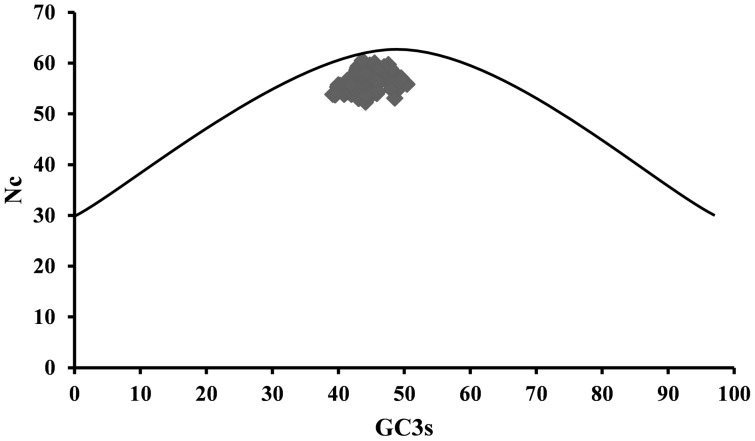
N_c_ of codons used plotted against the GC3s. The continuous curve as seen in the plot represents the relationship between GC3s and the N_c_ in the absence of selection. All the values lie below the expected curve. The curve represents the expected codon usage if GC compositional constraints alone account for codon usage bias.

**Figure 3:**
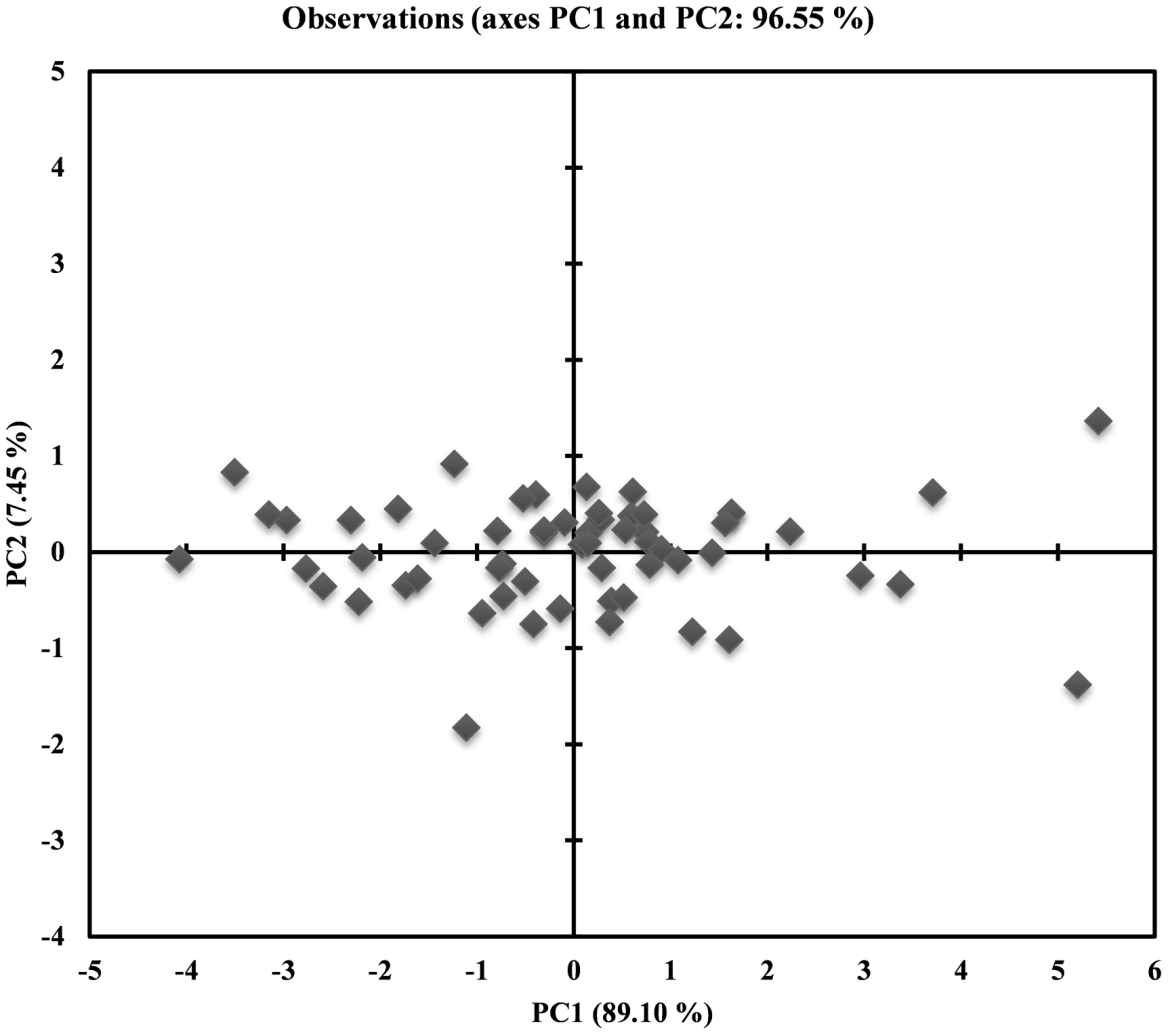
A Scatter plot of the optimal codon distribution on the first and second principal axes are derived from PCA analysis. The first axis (PC1) explained 89.90% of total variance, while the second axis (PC2) accounted for 7.45% of total variance.

**Figure 4:**
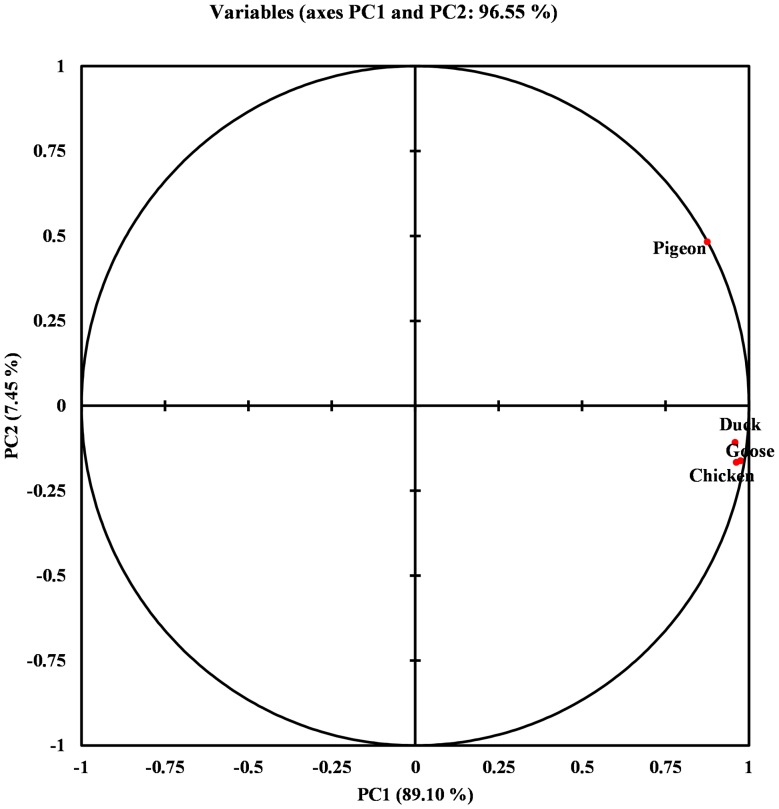
Relatedness among the species based on the codon usage. Chicken, Goose and Duck are more related in terms of codon bias than Pigeon which is an outlier.

The Nc value is plotted against the corresponding GC3s. The genes having their codon selection constrained to GC composition are supposed to lie on the continuous curve which represents a random codon usage. If the gene lies below the curve it represents mutational bias and translational selection. All the points were found to lie just below the curve (Figure 2). Furthermore, a significant positive correlation between the values of GC3s and Nc was observed. There was almost no difference between the CAI values for all the isolates. The average CAI was found to be 0.174 (p <0.05), which is apparently low as CAI ranges from 0 to 1. Principal component analysis using Pearson correlation was performed to evaluate the relationship between the first two axes of PCA, GRAVY, aromaticity, GC3s and aliphatic index (Table 4). The GRAVY, aromaticity, GC3s were found to have no correlation with both axes. To represent the variation in position of each codon a scatter plot for the optimal codon usage is plotted between the PC1 and PC2 (Figure 3). The PC1 accounts for 89.10% and PC2 accounts for 7.45% of the total variation. Thus the first axis accounts for the major impact on total variation in synonymous codon usage as compared to an appreciable impact by the second axis. The correlation between the isolates from four species is represented in the plot of isolates against the PC1 and PC2 (Figure 4).

**Table 4:** Summary of correlation analysis between the first two axes in COA, GC3s, GRAVY, aromaticity and aliphatic Index (AI) in the selected complete fusion (F) gene sequences for Newcastle disease virus isolates from four major avian species.

		GRAVY	Aromaticity	GC3s	Aliphatic Index
Axis 1	r	−0.531	0.379	−0.576	−0.780
	p	0.469	0.621	0.424	0.220
Axis 2	r	0.594	−0.332	0.617	0.815
	p	0.406	0.668	0.383	0.185

No correlation was observed between the two axes, GRAVY, aromaticity, GC3s and AI.

*P*-value ≤ 0.05; *P*-value ≤ 0.01 were used for the correlation analysis.

### Phylogenetic analysis

**Figure 5:**
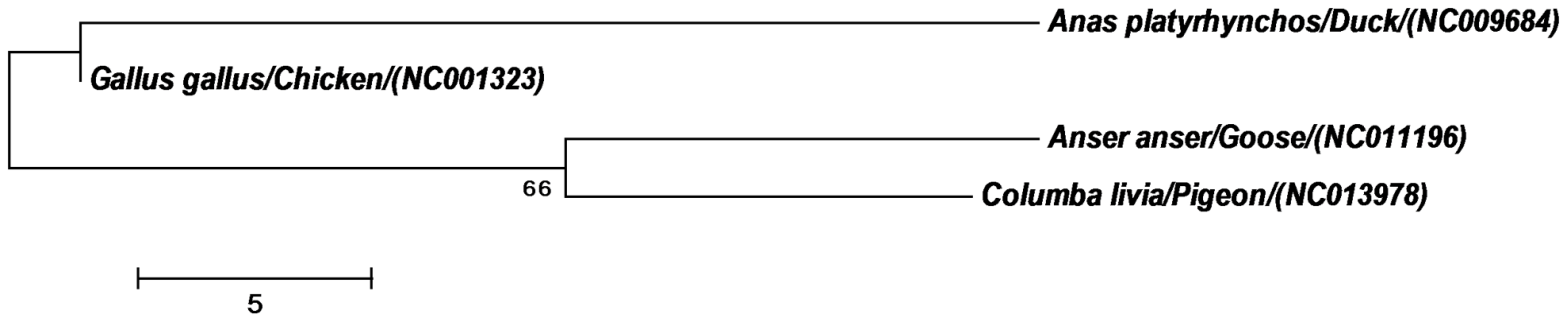
Phylogenetic tree illustrating relationship among the four host species following the neighbour-joining method using MEGA6 software. Parameters include: pairwise deletion, 1000 replicates for bootstrap analysis and Dayhoff substitution model. The reference sequence of the mitochondrial DNA was taken to study the relationship among the host species.

The phylogenetic tree of the major avian host species of NDV represents the relationship between them. It was observed that *Anas platyrhynchos* (Duck) and *Gallus gallus* (Chicken) are ancestrally more correlated whereas; *Anser anser* (Goose) and *Columba livia* (Duck) bear a closer ancestral relationship (Figure 5).

**Figure 6:**
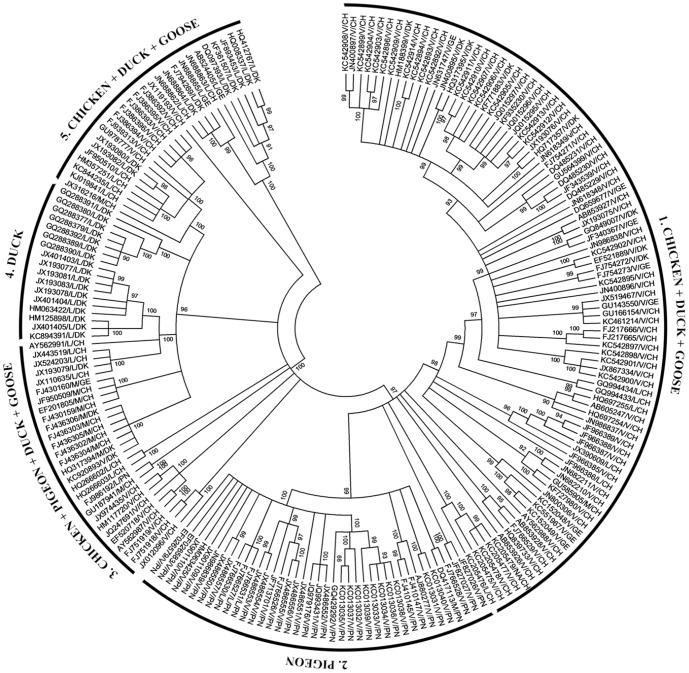
Phylogenetic tree illustrating relationship among the 201 Newcastle disease virus (NDV) strain (labelled as accession no/pathogenic/species) following the neighbour-joining method using MEGA6 software. Parameters include: pairwise deletion, 1000 replicates for bootstrap analysis and Jukes-Cantor substitution model, the rate variation among sites was modelled with a gamma distribution (shape parameter  =  5). L, M and V stands for lentogenic, mesogenic and velogenic strains. CH, PN, DK and GE stands for the chicken, pigeon, duck and goose, respectively.

The phylogenetic tree of the 201 NDV isolates represents the relationship which can be categorized on two bases (Figure 6). The first basis is being species from which the strain was isolated and the second being virulence shown in that species based on the F protein cleavage site. It was observed that on the basis of species except one pigeon isolate (FJ986192) lying in region 3 all the pigeon isolates were seen to lie in region 2 (Figure 6). The region 1 and 5 consisted of isolates from chicken, duck and goose whereas; the region 4 consisted of isolates of only duck. It is evident from the phylogenetic tree that isolates from pigeon clearly out lies and is not found mixed as is seen in case of chicken, duck and goose. A group of isolates from duck is also seen in region 4 to out lie from the rest. On the basis of virulence the phylogenetic tree can be seen to represent a similar trend, all the isolates from duck species lying in region 4 are lysogenic. Most of the mesogenic isolates from chicken are seen to lie in region 3. Very few reported strains from pigeon are lentogenic and mesogenic whereas most are velogenic and seen to group together in region 2. The region 1 comprises of most of the velogenic isolates of chicken, goose and duck.

## Discussion

NDV is one of the most important diseases of poultry and is endemic in many parts of the world. Occasionally the virus has also been reported from many different animal species. The F protein determines the extent of infectivity of NDV [3], [5]. Although the major players of NDV virulence and pathogenicity are F and HN, but its gradients are largely multigenic. The F being the major antigenic determinant in NDV changed our views of considering HN as a major protective antigen [19]. The interaction between the host cell membrane and the fusion protein may depend on the type of species that gets infected with NDV. It has been shown that mutational pressure plays an important role in codon usage bias in NDV [42]. The codon bias in the F protein may vary within the species that are infected with NDV thus it is significant to address codon bias in F protein of NDV. Although NDV has been isolated from many other avian as well as non-avian hosts but, their meager number in the GenBank makes the data insufficient to consider for the present study. Four major avian species, namely chicken, duck, pigeon and goose were chosen for the present study considering the fact that most of the NDV strains are isolated from these species. Although only few NDV sequences are reported from goose, a total of 201 GenBank entries were included in the study covers the major isolates from four avian species.

There is an obvious difference between these values for the isolates with lower Nc values suggesting that a lower Nc represents greater bias in codon usage. Amongst the four major host species maximum bias is for isolate from goose and least for isolate from duck. Although, there is a clear variation in the codon usage by the isolates from the four species still similarities within isolates can be observed. A plot of Nc values against GC3s effectively demonstrates heterogeneity [31].

The results suggest that the GC mutational bias is maximum in case of isolates from goose and minimum in case of isolates from duck. Low value of CAI obtained after analyzing all the isolates from the four species suggest lower codon usage bias and its low expression level. The frequencies of aromatic and aliphatic amino acids were found to have no association with the variation in codon usage in the F gene. Our results showed that the relatedness between the isolates from four species can be grouped according to the minimum positional variation. The isolates from chicken, goose and duck have least variation and can be considered as closely related isolates in terms of codon usage. In contrast, marked variation among isolates from pigeon suggests their distance in terms of codon usage. It corroborates with the fact that greater the distance between the isolates, greater is the variation in codon usage. It is also evident from the phylogenetic tree of NDV that isolates of pigeon clearly lies separately. Moreover, some of the isolates from duck also lie separately. Thus, the phylogenetic tree (Figure 6) and the relatedness between the species based on codon usage bias (Figure 4) clearly complement each other. The relationship between the host species and the NDV isolates from the host does not represent a significant correlation in our study. To the best of our understanding the present work is the most comprehensive codon bias analysis of a viral protein from species' point of view. It would be interesting to statistically investigate the NPL complex of NDV in terms of its codon usage and its role in virulence if any.

## References

[1] Lamb RA, Collins PL, Kolakofsky D, Melero JA, Nagai Y, et al. (2005) Family Paramyxoviridae. In: Fauquet, C.M. (Ed.), Virus Taxonomy: The Classification and Nomenclature of Viruses. The Eighth Report of the International Committee in Taxonomy of Viruses. Press EAeditor.

[2] Sharma B, Pokhriyal M, Rai GK, Saxena M, Ratta B, et al (2012) Isolation of Newcastle disease virus from a non-avian host (sheep) and its implications. Arch Virol 157:1565–1567. 22543636 10.1007/s00705-012-1317-8

[3] Panda A, Huang Z, Elankumaran S, Rockemann DD, Samal SK (2004) Role of fusion protein cleavage site in the virulence of Newcastle disease virus. Microb Pathog 36:1–10. 14643634 10.1016/j.micpath.2003.07.003 PMC7125746

[4] Samal S, Kumar S, Khattar SK, Samal SK (2011) A single amino acid change, Q114R, in the cleavage-site sequence of Newcastle disease virus fusion protein attenuates viral replication and pathogenicity. J Gen Virol 92:2333–2338. 21677091 10.1099/vir.0.033399-0

[5] Peeters BP, de Leeuw OS, Koch G, Gielkens AL (1999) Rescue of Newcastle disease virus from cloned cDNA: evidence that cleavability of the fusion protein is a major determinant for virulence. J Virol 73:5001–5009. 10233962 10.1128/jvi.73.6.5001-5009.1999 PMC112544

[6] Romer-Oberdorfer A, Werner O, Veits J, Mebatsion T, Mettenleiter TC (2003) Contribution of the length of the HN protein and the sequence of the F protein cleavage site to Newcastle disease virus pathogenicity. J Gen Virol 84:3121–3129. 14573818 10.1099/vir.0.19416-0

[7] Morrison TG (2003) Structure and function of a paramyxovirus fusion protein. Biochim Biophys Acta 1614:73–84. 12873767 10.1016/s0005-2736(03)00164-0

[8] Glickman RL, Syddall RJ, Iorio RM, Sheehan JP, Bratt MA (1988) Quantitative basic residue requirements in the cleavage-activation site of the fusion glycoprotein as a determinant of virulence for Newcastle disease virus. J Virol 62:354–356. 3275436 10.1128/jvi.62.1.354-356.1988 PMC250538

[9] Toyoda T, Sakaguchi T, Imai K, Inocencio NM, Gotoh B, et al (1987) Structural comparison of the cleavage-activation site of the fusion glycoprotein between virulent and avirulent strains of Newcastle disease virus. Virology 158:242–247. 3576973 10.1016/0042-6822(87)90261-3

[10] Kattenbelt JA, Stevens MP, Gould AR (2006) Sequence variation in the Newcastle disease virus genome. Virus Res 116:168–184. 16430984 10.1016/j.virusres.2005.10.001

[11] Nagai Y, Klenk HD, Rott R (1976) Proteolytic cleavage of the viral glycoproteins and its significance for the virulence of Newcastle disease virus. Virology 72:494–508. 948870 10.1016/0042-6822(76)90178-1

[12] Samal SK, editor (2011) Newcastle disease and related avian paramyxoviruses. Norfolk, United Kingdom: Caister Academic Press. 69–114 p.

[13] Samal S, Khattar SK, Kumar S, Collins PL, Samal SK (2012) Coordinate deletion of N-glycans from the heptad repeats of the fusion F protein of Newcastle disease virus yields a hyperfusogenic virus with increased replication, virulence, and immunogenicity. J Virol 86:2501–2511. 22205748 10.1128/JVI.06380-11 PMC3302274

[14] Samal S, Khattar SK, Paldurai A, Palaniyandi S, Zhu X, et al (2013) Mutations in the cytoplasmic domain of the Newcastle disease virus fusion protein confer hyperfusogenic phenotypes modulating viral replication and pathogenicity. J Virol 87:10083–10093. 23843643 10.1128/JVI.01446-13 PMC3754023

[15] Sakaguchi T, Fujii Y, Kiyotani K, Yoshida T (1994) Correlation of proteolytic cleavage of F protein precursors in paramyxoviruses with expression of the fur, PACE4 and PC6 genes in mammalian cells. J Gen Virol 75 (Pt 10):2821–2827. 7931173 10.1099/0022-1317-75-10-2821

[16] Nagai Y (1995) Virus activation by host proteinases. A pivotal role in the spread of infection, tissue tropism and pathogenicity. Microbiol Immunol 39:1–9. 7783672 10.1111/j.1348-0421.1995.tb02161.x

[17] Wakamatsu N, King DJ, Seal BS, Peeters BP, Brown CC (2006) The effect on pathogenesis of Newcastle disease virus LaSota strain from a mutation of the fusion cleavage site to a virulent sequence. Avian Dis 50:483–488. 17274282 10.1637/7515-020706R.1

[18] Cornax I, Diel DG, Rue CA, Estevez C, Yu Q, et al (2013) Newcastle disease virus fusion and haemagglutinin-neuraminidase proteins contribute to its macrophage host range. J Gen Virol 94:1189–1194. 23426356 10.1099/vir.0.048579-0 PMC3709627

[19] Kumar S, Nayak B, Collins PL, Samal SK (2011) Evaluation of the Newcastle disease virus F and HN proteins in protective immunity by using a recombinant avian paramyxovirus type 3 vector in chickens. J Virol 85:6521–6534. 21525340 10.1128/JVI.00367-11 PMC3126493

[20] Alexander DJ (1998) Newcastle disease and other avian paramyxoviruses. University of Pennsylvania, Kennett Square, PA: American Association of Avian Pathologists.

[21] Lloyd AT, Sharp PM (1992) Evolution of codon usage patterns: the extent and nature of divergence between Candida albicans and Saccharomyces cerevisiae. Nucleic Acids Res 20:5289–5295. 1437548 10.1093/nar/20.20.5289 PMC334333

[22] Botzman M, Margalit H (2011) Variation in global codon usage bias among prokaryotic organisms is associated with their lifestyles. Genome Biol 12:R109. 22032172 10.1186/gb-2011-12-10-r109 PMC3333779

[23] Grantham R, Gautier C, Gouy M, Mercier R, Pave A (1980) Codon catalog usage and the genome hypothesis. Nucleic Acids Res 8:r49–r62. 6986610 10.1093/nar/8.1.197-c PMC327256

[24] Marin A, Bertranpetit J, Oliver JL, Medina JR (1989) Variation in G + C-content and codon choice: differences among synonymous codon groups in vertebrate genes. Nucleic Acids Res 17:6181–6189. 2570402 10.1093/nar/17.15.6181 PMC318270

[25] Stoletzki N, Eyre-Walker A (2007) Synonymous codon usage in Escherichia coli: selection for translational accuracy. Mol Biol Evol 24:374–381. 17101719 10.1093/molbev/msl166

[26] Rao Y, Wu G, Wang Z, Chai X, Nie Q, et al (2011) Mutation bias is the driving force of codon usage in the Gallus gallus genome. DNA Res 18:499–512. 22039174 10.1093/dnares/dsr035 PMC3223081

[27] Wang M, Zhang J, Zhou JH, Chen HT, Ma LN, et al (2011) Analysis of codon usage in bovine viral diarrhea virus. Arch Virol 156:153–160. 21069395 10.1007/s00705-010-0848-0 PMC7087306

[28] Zhang J, Wang M, Liu WQ, Zhou JH, Chen HT, et al (2011) Analysis of codon usage and nucleotide composition bias in polioviruses. Virol J 8:146. 21450075 10.1186/1743-422X-8-146 PMC3079669

[29] Liu X, Wu C, Chen AY (2010) Codon usage bias and recombination events for neuraminidase and hemagglutinin genes in Chinese isolates of influenza A virus subtype H9N2. Arch Virol 155:685–693. 20300785 10.1007/s00705-010-0631-2

[30] Sharp PM, Li WH (1986) Codon usage in regulatory genes in Escherichia coli does not reflect selection for 'rare' codons. Nucleic Acids Res 14:7737–7749. 3534792 10.1093/nar/14.19.7737 PMC311793

[31] Wright F (1990) The 'effective number of codons' used in a gene. Gene 87:23–29. 2110097 10.1016/0378-1119(90)90491-9

[32] Comeron JM, Aguade M (1998) An evaluation of measures of synonymous codon usage bias. J Mol Evol 47:268–274. 9732453 10.1007/pl00006384

[33] Duret L, Mouchiroud D (1999) Expression pattern and, surprisingly, gene length shape codon usage in Caenorhabditis, Drosophila, and Arabidopsis. Proc Natl Acad Sci U S A 96:4482–4487. 10200288 10.1073/pnas.96.8.4482 PMC16358

[34] Coghlan A, Wolfe KH (2000) Relationship of codon bias to mRNA concentration and protein length in Saccharomyces cerevisiae. Yeast 16:1131–1145. 10953085 10.1002/1097-0061(20000915)16:12<1131::AID-YEA609>3.0.CO;2-F

[35] Sharp PM, Li WH (1987) The codon Adaptation Index-a measure of directional synonymous codon usage bias, and its potential applications. Nucleic Acids Res 15:1281–1295. 3547335 10.1093/nar/15.3.1281 PMC340524

[36] Ikai A (1980) Thermostability and aliphatic index of globular proteins. J Biochem 88:1895–1898. 7462208

[37] Kyte J, Doolittle RF (1982) A simple method for displaying the hydropathic character of a protein. J Mol Biol 157:105–132. 7108955 10.1016/0022-2836(82)90515-0

[38] Zhang Y, Liu Y, Liu W, Zhou J, Chen H, et al (2011) Analysis of synonymous codon usage in hepatitis A virus. Virol J 8:174. 21496278 10.1186/1743-422X-8-174 PMC3087699

[39] Reyes A, Pesole G, Saccone C (1998) Complete mitochondrial DNA sequence of the fat dormouse, Glis glis: further evidence of rodent paraphyly. Mol Biol Evol 15:499–505. 9580978 10.1093/oxfordjournals.molbev.a025949

[40] Pollock DD, Eisen JA, Doggett NA, Cummings MP (2000) A case for evolutionary genomics and the comprehensive examination of sequence biodiversity. Mol Biol Evol 17:1776–1788. 11110893 10.1093/oxfordjournals.molbev.a026278

[41] Ikemura T (1981) Correlation between the abundance of Escherichia coli transfer RNAs and the occurrence of the respective codons in its protein genes. J Mol Biol 146:1–21. 6167728 10.1016/0022-2836(81)90363-6

[42] Cao HW, Li DS, Zhang H (2014) Analysis of synonymous codon usage in Newcastle disease virus hemagglutinin-neuraminidase (HN) gene and fusion protein (F) gene. Virusdisease 25:132–136. 24426322 10.1007/s13337-013-0175-7 PMC3889239

